# Editorial: An update on brassinosteroids: homeostasis, crosstalk, and adaptation to environmental stress, Volume II

**DOI:** 10.3389/fpls.2023.1194566

**Published:** 2023-04-18

**Authors:** Damian Gruszka, Andrzej Bajguz, Qian-Feng Li, Shamsul Hayat, Mats Hansson

**Affiliations:** ^1^ Institute of Biology, Biotechnology and Environmental Protection, Faculty of Natural Sciences, University of Silesia, Katowice, Poland; ^2^ Faculty of Biology, University of Bialystok, Bialystok, Poland; ^3^ Jiangsu Key Laboratory of Crop Genomics and Molecular Breeding, College of Agriculture, Yangzhou University, Yangzhou, Jiangsu, China; ^4^ Faculty of Life Sciences, Department of Botany, Aligarh Muslim University, Aligarh, India; ^5^ Department of Biology, Lund University, Lund, Sweden

**Keywords:** brassinosteroid biosynthesis, brassinosteroid signalling, hormonal homeostasis, signalling crosstalk, brassinosteroid-mediated stress responses

## Introduction

Since the discovery of brassinosteroids (BRs) in the 1970’s, these steroid phytohormones have been proven to be crucial regulators of various aspects of plant biology, from germination to leaf senescence, reproductive development, and stress responses. Molecular mechanisms regulating the BR homeostasis, signaling, and interactions with other phytohormones were described to a significant degree, particularly in the model plant species *Arabidopsis thaliana*. However, our knowledge about these aspects in other species, including crops, is much more limited. Therefore, the Research Topic ‘*An Update on Brassinosteroids: Homeostasis, Crosstalk, and Adaptation to Environmental Stress, Volume II*’ introduces the latest findings in the regulation of BR signaling, its interconnection with the phytohormonal and stress signaling pathways, and the role of BRs in plant adaptation to environmental conditions. This Research Topic includes five research papers and one review article.

In the research article by Song et al., the authors identified 444 genes encoding LEUCINE-RICH REPEAT RECEPTOR-LIKE PROTEIN KINASES (LRR-RLKs) in the genome of *Brassica napus* (rapeseed). The *LRR-RLK* family members play crucial roles during plant development and reaction to environmental conditions. Interestingly, the identified *LRR-RLK* genes showed unique expression profiles in 12 vegetative and reproductive tissues, as well as during response to abiotic stresses. In a separate experiment, partial knockouts of six BR receptor genes *BRASSINOSTEROID INSENSITIVE1* (*BnBRI1*) were generated through the CRISPR/Cas9 approach. Noteworthy, one of the produced semi-dwarf lines did not show a decrease in yield when compared with a reference genotype. This study provided an insight into the LRR-RLK family in *B. napus* and supplemented germplasm of this important crop with the new semi-dwarf line which may be an alternative in breeding programmes.

In another research article by Shuai et al., a tomato (*Solanum lycopersicum*) orthologue of the *CESTA* (*CES*) gene, which encodes a transcription factor belonging to the BR ENHANCED EXPRESSION (BEE) subfamily, was identified. In Arabidopsis, the CES transcription factor and other members of the BEE subfamily stimulate expression of various BR-responsive genes. In this study, an effect of the *SlCES* gene overexpression was analyzed. The experiments indicated that the function of *SlCES* in mediating the BR-dependent gene expression is conserved in tomato, and its overexpression may also regulate homeostasis of gibberellins. Noteworthy, tomato lines in which the *SlCES* gene was overexpressed showed increased chilling tolerance (both at the vegetative and reproductive stage of development) and altered fruit characteristics (including increased fruit weight and calcium accumulation). Interestingly, the fruits of the overexpression line produced more, but smaller seeds.

The function of another member of the BEE subfamily (*GhBEE3-LIKE* gene) in the regulation of drought tolerance in cotton (*Gossypium hirsutum*) was studied by Chen et al. Analysis of the *GhBEE3-LIKE* gene expression pattern indicated that this gene plays a role in shoot growth and development. In cotton, the *GhBEE3-LIKE* gene is repressed by BR and drought treatments. Moreover, cotton plants in which the *GhBEE3-LIKE* gene was knocked down were more tolerant to drought. This indicates that the *GhBEE3-LIKE* gene may negatively regulate drought tolerance in cotton. The results of this study suggest that the BR-induced inhibition of the *GhBEE3-LIKE* gene promotes the expression of stress-related genes to stimulate drought tolerance.

In a study by Pantoja-Benavides et al., the effect of foliar applications of cytokinins (CK) or BR on acclimatization of rice (*Oryza sativa*) plants to heat stress was analyzed. Rice plants treated with three foliar sprays of CK or BR during the heat stress period showed increased stomatal conductance and lower leaf temperature when compared to heat-stressed control plants. It was shown that three applications of CK or BR mitigated the heat stress effects. Additionally, the foliar applications of CK or BR at the flowering and grain-filling stages during the stress conditions increased the chlorophyll content and fluorescence parameters. The CK or BR applications also improved panicle characteristics (number of filled spikelets and percentage of panicle blanking). The results of this study led to the conclusion that foliar applications of BR or CK can be considered as a strategy to alleviate the effect of heat stress on the physiological parameters of rice at different stages of development.

An effect of exogenous BRs on the response of mini Chinese cabbage (*Brassica rapa ssp. pekinensis*) plants to calcium deficiency, which is manifested by tip-burn, was studied in the report by Li et al. The calcium deficiency is the major cause of tip-burn and may lead to serious yield loss. It was reported that exogenous BRs reduced the tip-burn incidence rate and disease index. Interestingly, the exogenous BRs increased the contents of cellulose, hemifiber, and water-soluble pectin in stress-treated plants, which contributed to maintaining the stability of cell wall structure. The results reported in this study suggested that the exogenous BRs mitigate the tip-burn incidence caused by the calcium deficiency by maintaining higher chlorophyll content and photosynthesis rate. These observations may help with reducing the occurrence of calcium deficiency-related tip-burn during mini Chinese cabbage cultivation.

In the review article by Zolkiewicz and Gruszka, the authors provided a comprehensive and detailed description of the GLYCOGEN SYNTHASE KINASES (GSKs) and their multiple roles in the regulation of development, stress responses, and reproduction in model and crop plant species. The best known representative of this family in Arabidopsis, BRASSINOSTEROID INSENTISIVE2 (BIN2/SK21), was initially characterized as a negative regulator of BR signaling. However, further investigations implicated the GSK/SK proteins in signaling pathways of other phytohormones and stress-response processes. Thus, the GSK/SK proteins are currently recognized as hubs interconnecting various signaling pathways and modulators of plant development, responses to environmental cues, and reproduction. Apart from highlighting the role of the GSK/SK proteins in BR signaling, the review provides a detailed description of mechanisms which regulate stability and activity of the GSK/SK proteins and their subcellular localization during plant development and adaptations to environmental conditions. Additionally, the involvement of the GSK/SK proteins in signaling pathways regulating diverse physiological processes, responses to biotic and abiotic stresses, and plant reproduction was also extensively illustrated and described in this review.

This Research Topic provided novel and interesting insight into the mechanisms regulating BR signaling, as well as BR-dependent gene expression and stress responses. However, further research is needed to shed light on other important aspects, such as the function of microRNAs in the regulation of BR homeostasis and the role of BR in epigenetic regulation of gene expression.

## Author contributions

DG wrote the manuscript. All authors revised the manuscript and gave final approval for publication.

